# The Protective Effect of Citronellol against Doxorubicin-Induced Cardiotoxicity in Rats

**DOI:** 10.3390/biomedicines11102820

**Published:** 2023-10-18

**Authors:** Sania Munir, Rizwan Hafeez, Waqas Younis, Muhammad Nasir Hayat Malik, Muhammad Usman Munir, Wajiha Manzoor, Muryam Abdul Razzaq, Luciane Barbosa Pessoa, Katiana Simões Lopes, Francislaine Aparecida dos Reis Lívero, Arquimedes Gasparotto Junior

**Affiliations:** 1 The Faculty of Pharmacy, Superior University, Lahore 54000, Pakistan; raosanu1994@gmail.com; 2 Faculty of Pharmaceutical Sciences, University of Central Punjab, Lahore 54000, Pakistan; rizwanhafeez128@gmail.com; 3 Department of Pharmacology, Faculty of Pharmacy, The University of Lahore, Lahore 54590, Pakistan; nasir.hayat@pharm.uol.edu.pk (M.N.H.M.); wajeehamanzoor4@gmail.com (W.M.); muryam_ar@yahoo.com (M.A.R.); 4 Division of Endocrinology, Diabetes and Metabolism, Department of Medicine, New York University Grossman School of Medicine, 550 Ist Ave, New York, NY 10016, USA; 5 Australian Institute for Bioengineering & Nanotechnology, The University of Queensland, Brisbane, QLD 4072, Australia; rph.usman@gmail.com; 6 Laboratory of Cardiovascular Pharmacology (LaFac), Faculty of Health Sciences, Federal University of Grande Dourados, Dourados 79804-970, MS, Brazil; lbpessoa02@gmail.com (L.B.P.); kati_sims@hotmail.com (K.S.L.); 7 Laboratory of Cardiometabolic Pharmacology, Department of Pharmacology, Federal University of Parana, Curitiba 81530-900, PR, Brazil; francislaine@ufpr.br

**Keywords:** anti-inflammatory, antioxidant, heart attack

## Abstract

Citronellol has been reported to have anti-inflammatory, anti-cancer, and antihypertensive activities, but its effect on myocardial ischemia is still unclear. The aim of this study was to investigate the therapeutic effects and pharmacological mechanisms of citronellol on ischemia. Therefore, a rat model of myocardial ischemia was established using the doxorubicin (DOX) model. To induce cardiotoxicity, the rats were given DOX (2.5 mg/kg) intraperitoneally over a 14-day period. Group I served as the control and received tween 80 (0.2%), group II received the vehicle and DOX, group III received the standard drug dexrazoxane and DOX, whereas groups IV, V, and VI were treated orally with citronellol (25, 50, and 100 mg/kg) and DOX, respectively. After treatment, the rats were euthanized, and blood samples were collected to assess the levels of serum cardiac markers, lipid profiles, and tissue antioxidant enzymes. The gene expressions of eNOS, PPAR-g, IL-10, VEGF, and NFkB-1 were also determined using real-time polymerase chain reactions. Simultaneous treatment with DOX and citronellol reduced cardiac antioxidant enzymes and lipid biomarkers in a dose-dependent manner. Citronellol also increased the expression of anti-inflammatory cytokines while reducing the expression of pro-inflammatory cytokines. Therefore, it can be concluded that citronellol may have potential cardioprotective effects in preventing DOX-induced cardiotoxicity.

## 1. Introduction

Cardiovascular diseases (CVDs), specifically ischemic heart diseases (IHDs), are increasing in their impacts on health and mortality worldwide [1]. IHDs, also referred to as coronary artery diseases, are major contributors to health issues and deaths worldwide. These conditions occur when the coronary arteries, responsible for supplying blood to the heart muscle, become narrow or blocked due to plaque buildup. As a result, blood flow and oxygen supply to the heart are reduced, leading to various complications related to the cardiovascular system. Morbidities associated with IHD include angina (chest pain or discomfort), heart attacks, heart failure, and arrhythmias. These conditions significantly affect a person’s quality of life and ability to function [2]. IHD affects approximately 126 million individuals globally (1655 per 100,000), which accounts for approximately 1.72% of the world’s population. In 2017, IHD was responsible for nine million reported deaths [3]. The World Health Organization (WHO) estimates that CVDs are the cause of 80% of all deaths, with developing countries carrying 86% of the global burden associated with these diseases.

The initial event in ischemic heart disease is typically the disruption of blood flow to a specific region. This can occur due to a blood clot forming within a blood vessel (thrombus), or because of a blockage caused by atherosclerosis (a buildup of cholesterol and other substances in the walls of the artery). Other causes can include sudden contraction of the blood vessel (vasospasm) or obstruction caused by a detached blood clot or foreign object (embolism). Reactive oxygen species (ROS), inflammation, and calcium overload play important roles in the development of ischemia [4]. Various inflammatory and pro-inflammatory cytokines, such as nuclear factor kappa-B (NF-κB) and interleukin 10 (IL-10), regulate arterial pressure and assist in cardiac remodeling [2]. PPARγ and IL-10 help reduce inflammation in vascular smooth muscle and cardiac tissue by inhibiting other pro-inflammatory cytokines, monocytes, and macrophage activation, while NF-κB promotes inflammation and cardiac toxicity [5]. During times of inadequate nutrient supply and low oxygen levels (hypoxia), vascular endothelial growth factor (VEGF-A) also plays a crucial role in protecting the heart by inducing the growth of new blood vessels (angiogenesis) and collateral development of coronary arteries [6].

Understanding how ischemia develops helps in creating strategies to minimize tissue damage and aid in recovery. Furthermore, addressing underlying risk factors, such as managing high blood pressure, controlling cholesterol levels, and promoting a healthy lifestyle, can help prevent or lower the risk of ischemic events. Taking timely action, like restoring blood flow through procedures such as thrombolysis, angioplasty, or bypass surgery, can greatly improve outcomes in ischemic conditions. However, although treatment that restores blood flow to ischemic tissues may seem like a logical therapy, it can actually worsen cardiac injury by increasing ROS production [7].

Phytochemicals are natural compounds that occur in plants and have been found to have various properties that promote good health. These compounds are known for their antioxidant properties, which help protect our cells from damage caused by free radicals. They may also have anti-inflammatory effects and contribute to a strong immune system. Some examples of phytochemicals include flavonoids, carotenoids, and polyphenols, which can be found in fruits, vegetables, whole grains, legumes, herbs, and spices. Therefore, phytochemicals with antioxidant and anti-inflammatory activities are potential candidates for use as agents that protect the heart [8].

Citronellol is a natural compound that can be found in many plants, particularly in certain varieties of citrus fruits like lemons and oranges. It is also present in other plants such as roses, geranium, and lavender [9]. Citronellol is known for its pleasant floral aroma and is commonly used in perfumes, cosmetics, and personal care products. In terms of chemistry, citronellol is classified as a monoterpene alcohol and has the molecular formula C_10_H_20_O [10]. Numerous studies have shown that citronellol has antioxidant [9], anti-inflammatory [10], neuroprotective [11], anti-diabetic [12], anti-cancer [13], and antihypertensive [14] effects. The volatile oil extracted from *Cymbopogon winterianus* Jowitt ex Bor, which is the primary source of citronellol, has been reported to have significant antihypertensive, vasorelaxant [15,16], antioxidant, and anti-inflammatory [17] effects. Considering the potential pharmacological properties of citronellol against oxidative stress, inflammation, and hypertension, the purpose of this study is to evaluate the cardioprotective activity of citronellol in rats with doxorubicin-induced myocardial ischemia.

## 2. Materials and Methods

### 2.1. Chemicals

The following items were purchased from Sigma Aldrich Darmstadt for the current study: citronellol, doxorubicin (DOX), dexrazoxane, phosphate buffer, hydrogen peroxide (H_2_O_2_), trichloroacetic acid, sodium phosphate buffer, glacial acetic acid, n-butanol, and acetic acid. All of these items were of analytical grade.

### 2.2. Animals

Wistar rats of both sexes weighing between 150 and 200 g were acquired from the central animal facility at the University of Lahore. Upon their arrival, the animals were given a period of seven days to adjust to the laboratory conditions. During this time, they were kept in a controlled environment at a temperature of 26 ± 1 °C, with a 12 h light and dark cycle. Prior to conducting any of the planned studies, the researchers obtained proper authorization (#IREC-2020-87) from the University of Lahore Institutional Ethical Committee.

### 2.3. Experimental Design

After one week of acclimatization, the animals were divided into six groups, with each group containing six rats (n = 6). Myocardial ischemia was induced by administering DOX (2.5 mg/kg i.p.) through six injections on alternate days. The total cumulative dose given was 15 mg/kg [18]. Group I served as the control and received tween 80 (0.2%). The rats in group II received tween 80 (0.2%) along with DOX, while the rats in group III received the standard drug dexrazoxane (10:1 i.v.) in combination with DOX. The rats in groups IV, V, and VI received three doses of citronellol (25, 50, and 100 mg/kg; dissolved in tween 80 [0.2%]) along with DOX. All the treatments were administered via gavage for 14 days in conjunction with the DOX administration.

### 2.4. Serum Cardiac Markers and Lipid Profile

After the 14-day treatment period, all the animals were anaesthetized, and blood samples were taken. The serum was separated from the blood samples using centrifugation so that cardiac markers such as creatine kinase myocardial band (CK-MB), troponin I, lactate dehydrogenase (LDH), creatine phosphokinase (CPK), and serum glutamic-oxaloacetic transaminase (SGOT) could be measured [19]. Additionally, the levels of serum triglycerides (TG), total cholesterol (TC), high-density lipoprotein cholesterol (HDL-C), and low-density lipoprotein cholesterol were determined. All the markers were measured using an enzymatic colorimetric method.

### 2.5. Cardiac Tissue Redox Status

After obtaining the cardiac tissue samples, a 30% homogenate (weight/volume; containing 0.9% buffered KCl at pH 7.4) was acquired. The levels of superoxide dismutase (SOD), catalase (CAT), malondialdehyde (MDA), and glutathione (GSH) were determined using the techniques described by Marklund and Marklund in 1974 [19], Aebi in 1983 [20], Rotruck in 1973 [21], and Ellman in 1959 [22], respectively.

### 2.6. Quantitative Real-Time Polymerase Chain Reaction (qPCR)

The blood samples were also utilized for extracting RNA, from which we generated cDNA to examine the expression of the following genes: interleukin 10 (IL-10), nuclear factor kappa B subunit 1 (NFkB1), peroxisome proliferator-activated receptor gamma (PPARγ), vascular endothelial growth factor A (VEGFA), and endothelial nitric oxide synthase (eNOS) [23]. The gene sequencing employed for the primers is displayed in Table 1.

### 2.7. Statistical Analysis

The data were initially analyzed to determine if they had homogeneity of variance and a normal distribution. The data were then analyzed using a one-way analysis of variance (ANOVA) and Dunnett’s multiple comparison test. A significance level of 95% (*p* < 0.05) was chosen. The statistical analyses and graphs were generated using GraphPad Prism version 9.5.0. for macOS.

## 3. Results

### 3.1. Serum Cardiac Markers

**Figure 1:**
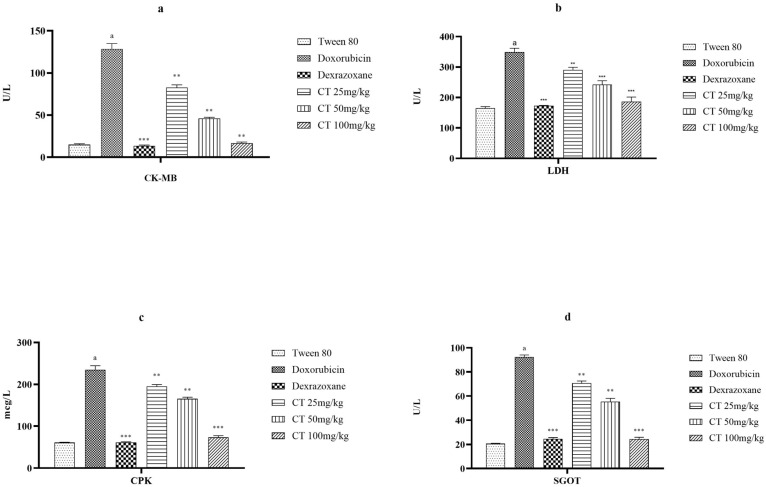
The impact of various groups on CK-MB (**a**), LDH (**b**), CPK (**c**), and SGOT (**d**) levels in DOX-intoxicated ischemic rats was examined. The values are presented as mean + SEM after performing a one-way ANOVA. Comparison of the experimental groups to the disease control group yielded a significance level of *p* < 0.001 denoted by *** and a significance level of *p* < 0.01 denoted by **. Additionally, when comparing the disease control group to the control group, there was a significant difference of *p* < 0.001 indicated by (a).

The impact of citronellol on various cardiac enzymes is illustrated in Figure 1. Doxorubicin considerably raised the amounts of LDH, CPK, and SGOT in the group affected by the disease compared to the group that received the vehicle. Administering citronellol at dosages of 25, 50, and 100 mg/kg reduced the levels of CK-MB, LDH, CPK, and SGOT in a manner that depended on the dose, compared to the group affected by the disease. Additionally, administering dexrazoxane reversed the increased levels of cardiac enzymes.

### 3.2. Lipid Profile

**Figure 2:**
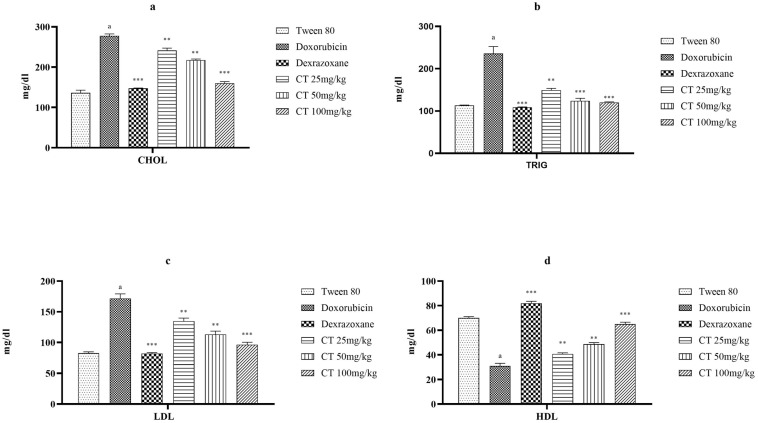
The impact of various groups on the levels of cholesterol (**a**), triglycerides (**b**), LDL (**c**), and HDL (**d**) in DOX-intoxicated ischemic rats. The values are represented as the mean plus the standard error of the mean (SEM) when a one-way ANOVA was applied. When comparing the experimental groups to the disease control group, *** represents a *p*-value of less than 0.001 and ** represents a *p*-value of less than 0.01. Additionally, when comparing the disease control group to the control group, (a) signifies a significant difference, with a *p*-value of less than 0.001.

In the group that received the DOX treatment, the levels of TC, TG, and LDL-C were significantly higher compared to the group that received only the vehicle control and rats treated with citronellol. On the other hand, citronellol significantly reduced the levels of TC, TG, and LDL compared to the group with the disease control, and for both TC and LDL, doses of 50 and 100 mg/kg showed similar results. However, the HDL levels were significantly lower in the DOX-treated group, while the use of citronellol at doses of 25, 50, and 100 mg/kg increased the HDL levels. Treatment with the standard drug dexrazoxane resulted in decreased TC and TG levels and increased HDL levels (Figure 2).

### 3.3. Cardiac Tissue Redox Status

**Figure 3:**
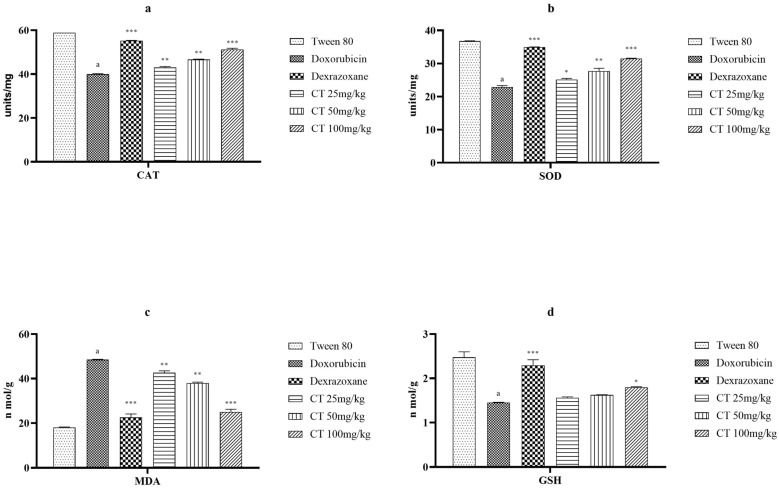
The impact of various groups on CAT (**a**), SOD (**b**), MDA (**c**), and GSH (**d**) levels in DOX-intoxicated ischemic rats was examined. The values are presented as the mean + SEM when a one-way ANOVA was utilized. In the comparisons between the experimental groups and the disease control group, *** denotes a significance level of *p* < 0.001, ** represents *p* < 0.01, and * indicates *p* < 0.05. Additionally, when comparing the disease control group with the control group, there was a significant difference observed in (a), with a value of *p* < 0.001.

The results depicted in Figure 3 demonstrate the influence of citronellol on CAT, SOD, MDA, and GSH. The administration of DOX led to a notable decrease in the levels of antioxidant enzymes. However, when citronellol was simultaneously administered, this effect was prevented. Citronellol particularly exhibited a significant impact on CAT and SOD levels. However, the effect of citronellol on GSH was not statistically significant at doses of 25 and 50 mg/kg, and only moderately effective at a dose of 100 mg/kg. Additionally, citronellol dose-dependently reduced MDA levels, particularly at doses of 50 and 100 mg/kg. Dexrazoxane, a standard drug, demonstrated similar effectiveness in restoring antioxidant enzyme levels to those observed in the control group.

### 3.4. Quantitative Real-Time Polymerase Chain Reaction (qPCR)

**Figure 4:**
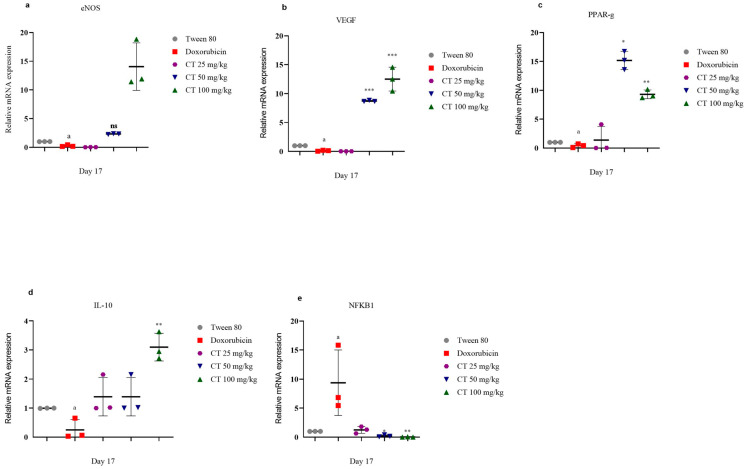
The effect of different groups on eNOS (**a**), VEGF (**b**), PPAR-g (**c**), IL-10 (**d**), and NFkB1 (**e**) in DOX-intoxicated ischemic rats was examined. Values are presented as mean plus standard error of the mean (SEM) when a one-way ANOVA was applied. When comparing the experimental groups to the disease control group, *** signifies a *p*-value of less than 0.001, ** signifies a *p*-value of less than 0.01, and * signifies a *p*-value of less than 0.05. When comparing the disease control group to the control group, there is a significant difference (a, *p*-value of less than 0.001) with regards to eNOS.

The group treated with DOX significantly decreased their expression of eNOS, PPAR-γ, IL-10, and VEGF compared to the group that received the vehicle control. On the other hand, the citronellol group showed an increase in the expression of eNOS in a manner that depended on the dose when compared to the group treated with DOX (Figure 4). Similarly, citronellol also increased the expression of IL-10, with the highest effect observed at a dosage of 100 mg/kg. Additionally, citronellol also enhanced the expression of VEGF at higher doses, specifically at 50 mg/kg and 100 mg/kg. The level of NFkB1 was significantly higher in the DOX-treated group compared to the group that received the vehicle control. All the dosages of citronellol resulted in significantly lower levels of NFkB1 compared to the DOX-treated group, in a manner that depended on the dose. The greatest reduction in NFkB1 was observed at a dosage of 100 mg/kg (Figure 4).

## 4. Discussion

Myocardial ischemia is a significant problem due to its high incidence and serious risk of developing severe heart diseases such as arrhythmia, heart failure, and heart attack [12]. Currently used synthetic drugs have unintended side effects and are used for long periods of time, leading to a shift towards the use of phytochemicals for treating cardiovascular diseases [24,25]. Among the various compounds used worldwide, citronellol deserves special attention. Citronellol is not only known for its pleasant scent but also for its potential antimicrobial, antioxidant, and anti-inflammatory properties. It can be used as a natural insect repellent and has been studied for its potential applications in fields such as food science, agriculture, and medicine. Building on previous preclinical studies, this study demonstrates that citronellol has the potential to protect the heart and prevent DOX-induced cardiotoxicity through its antioxidant and anti-inflammatory effects.

DOX-induced myocardial injury, also known as cardiotoxicity, is a well-known side effect of doxorubicin, a commonly used chemotherapy drug. Doxorubicin can cause damage to the heart muscle, leading to various cardiac complications. This can include the development of congestive heart failure, arrhythmias, and in severe cases, even a heart attack. Extensive biochemical changes were observed in the model of myocardial injury induced by doxorubicin. These changes indicate inflammation in the heart muscle and damage to the heart caused by reactive oxygen species (ROS) and the release of myocardial biomarkers into the bloodstream [26]. In this study, we observed that isolated treatment with doxorubicin increased the levels of cardiac enzymes, inflammatory biomarkers, as well as serum levels of ROS. CK-MB, LDH, SGOT, and CPK are biomarkers present in the blood that are used to evaluate the proper functioning of the heart [7]. The group of rats treated with doxorubicin showed increased levels of serum CK-MB, LDH, SGOT, and CPK, suggesting myocardial injury [27,28]. Treatment with citronellol significantly and dose-dependently lowered the levels of CK-MB, LDH, SGOT, and CPK. The decrease in these biomarkers in the rats treated with citronellol indicates its preventive role in maintaining the integrity of the membrane and protecting against damage caused by myocardial necrosis.

During myocardial ischemia, a limited oxygen supply leads to decreased energy production within the heart muscle cells. This imbalance between the production and clearance of ROS can cause an excessive buildup of these molecules. The increased presence of ROS can then lead to oxidative stress, which may contribute to further damage to the heart muscle during ischemia [4]. ROS play a significant role in the pathophysiology of myocardial ischemia by reducing sarcolemma calcium transport due to oxidative overload, which can increase intracellular Ca^2+^ overload and impair heart function [1]. Antioxidant enzymes such as CAT, GSH, and SOD have protective effects against injuries caused by free radicals by detoxifying them [29]. It was also observed that the levels of these enzymes were reduced in the rats treated with DOX, which indicates cardiac damage and oxidative stress. Similarly, increased levels of MDA suggest elevated lipid peroxidation in rats treated with DOX [28,29]. Treatment with citronellol mitigated the changes induced by DOX to lipid peroxidation and antioxidant enzyme levels. Significant increases in CAT, GSH, and SOD levels, as well as significant reductions in MDA serum levels, were observed. These findings suggest that treatment with citronellol improves the redox status and prevents heart damage caused by ROS.

VEGF stands for vascular endothelial growth factor. It is a protein called cytokine that is involved in cell signaling. VEGF plays a crucial role in the formation of new blood vessels, which is known as angiogenesis. VEGF has been linked to various diseases, including ischemic heart tissue, where it can contribute to the growth of new blood vessels that supply damaged heart muscles [6]. In the current study, the expression of the VEGF gene was significantly increased by citronellol, and similar results were found for eNOS. These findings suggest that the activation of endothelial nitric oxide synthase induced by vascular endothelial growth factor contributes to reducing the apoptosis of cardiac myocytes through an increase in nitric oxide synthesis. Under several conditions, the increase in nitric oxide availability contributes to the integrity of the vascular endothelium, resulting in vasodilation, a reduction in oxidative stress, and inhibition of local platelet aggregation [30,31]. Moreover, the activation of eNOS triggered by VEGF enhances the development of blood vessels in the heart during tissue ischemia [30,31]. Administering the human eNOS gene via gene therapy with adenovirus prior to a heart attack reduces damage to the heart tissue resulting from insufficient blood flow. This process increases the quantity of smaller vessels and improves heart contractility [32,33,34]. 

NF-κB, which stands for nuclear factor kappa-light-chain-enhancer of activated B cells, is a protein complex that plays a critical role in the immune system and cell signaling. It functions as a transcription factor, meaning it helps regulate the activity of genes involved in various cellular processes, including inflammation, immune response, and cell survival. NF-κB is commonly activated in response to pathogens, cytokines, and other stressors, and it is implicated in several diseases, such as cancer, autoimmune disorders, chronic inflammation, and ischemic injury [31]. In this study, the group treated with DOX exhibited significant inflammatory reactions in the myocardium, which also resulted in a significant increase in the expression of NF-κB in the myocardium. However, at a dosage of 100 mg/kg, citronellol significantly reduced the expression of NF-κB in the myocardium.

IL-10 is primarily known for its ability to reduce inflammation. It helps control the immune response by suppressing the activation of immune cells and reducing the production of substances that cause inflammation. This anti-inflammatory effect of IL-10 is important in preventing excessive immune responses and damage to tissues. Additionally, IL-10 has been found to support the growth and development of certain immune cells, like B-cells, and enhance their ability to produce antibodies. It also plays a role in maintaining the balance between different types of T-helper cells, promoting the development of regulatory T-cells that help suppress immune reactions. Overall, IL-10 acts as an important cytokine that helps regulate the immune system, ensuring a healthy balance and preventing excessive inflammation [5]. Moreover, IL-10 can prevent the harmful effects of inflammation on the heart and may protect against damage caused by inflammatory substances [5]. IL-10 also prevents macrophages from producing certain substances that contribute to inflammation, indicating its anti-inflammatory role. Citronellol, a substance, significantly increased the expression of IL-10, which suggests its potential in reducing inflammation and protecting the heart.

PPAR-γ, also known as peroxisome proliferator-activated receptor-gamma, is a protein that belongs to the nuclear receptor superfamily. It plays an important role in regulating gene expression patterns involved in various cellular functions, such as metabolism, inflammation, and adipogenesis [35]. PPAR-γ is also crucial in controlling genes related to the metabolic processes of the heart. If the PPAR-γ pathway is disrupted or impaired, it can result in changes in the heart’s structure and function, leading to cardiomyopathy. This condition is characterized by the enlargement, stiffening, or weakening of the heart muscle, affecting its ability to effectively pump blood. Thus, the decrease in PPAR-γ levels in the DOX-treated groups indicates a disturbance in lipid homeostasis and the induction of cardiomyopathy [35]. Low PPAR-γ levels increase fat storage in non-adipose tissue, causing lipotoxicity. Therefore, this lipid accumulation may be due to the downregulation of PPAR-γ [36]. The use of citronellol as a treatment resulted in a significant decrease in overall cholesterol, triglycerides, and low-density lipoprotein levels, while simultaneously increasing high-density lipoprotein levels. The groups treated with citronellol showed an increased expression of PPAR-γ levels, suggesting a positive impact on lipid metabolism.

There are two important limitations that are evident in our study. The data we obtained were unable to demonstrate the cumulative toxicity profile of citronellol, and its pharmacokinetic characteristics are unknown. It is crucial to comprehensively evaluate its safety and kinetic pattern before contemplating its extensive usage. This information can aid researchers and healthcare professionals in making informed decisions about its potential advantages and disadvantages.

## 5. Conclusions

The current study shows that citronellol effectively protects the heart in rats treated with doxorubicin. Citronellol exhibits antioxidant activity and potential for reducing inflammation by inhibiting the expression of genes that cause inflammation and stimulating genes that have anti-inflammatory effects. However, despite the promising nature of these findings, it is essential to assess the safety and pharmacokinetics of this substance before considering its potential for clinical use. This information can assist researchers and healthcare professionals in making well-informed decisions regarding the potential advantages and disadvantages of citronellol.

## Figures and Tables

**Table 1:** Primers used in PCR.

	Gene Name	Primers	Sequences (5′–3′)	BP	Temperature (°C)
1	*IL10*	IL10 Fw:	GCCCAGAAATCAAGGAGCATT	21	59
		IL10 Rv:	CAGCTGTATCCAGAGGGTCTTC	22	60
2	*NFkB1*	NFkB1 Fw:	CTGAGTCCCGCCCCTTCTAA	20	61
		NFkB1 Rv:	CCTCTGTGTAGCCCATCTGTC	21	60
3	*PPAR-γ*	PPAR-a Fw:	CCCTTTACCACGGTTGATTTCTC	23	60
		PPAR-a Rv:	CAGGCTCTACTTTGATCGCACT	22	60
4	*eNOS*	eNOS Fw:	TATTTGATGCTCGGGACTGCA	21	60
		eNOS Rv:	AAGATTGCCTCGGTTTGTTGC	21	60
5	*VEGFA*	VEGFA Fw:	GCCTCAGGACATGGCACTAT	20	59
		VEGFA Rv:	GGAGGAGGAGGAGCCATTAC	20	59

## Data Availability

The data presented in this study are available from the corresponding author upon request. The data are not publicly available due to privacy.

## References

[1] Bansal P., Gupta S.K., Ojha S.K., Nandave M., Mittal R., Kumari S. (2006). Cardioprotective effect of lycopene in the experimental model of myocardial ischemia-reperfusion injury. Mol. Cell Biochem..

[2] Song Q., Chu X., Zhang X., Bao Y., Zhang Y., Guo H. (2016). Mechanisms underlying the cardioprotective effect of Salvianic acid A against isoproterenol-induced myocardial ischemia injury in rats: Possible involvement of L-type calcium channels and myocardial contractility. J. Ethnopharmacol..

[3] Khan M.A., Hashim M.J., Mustafa H., Baniyas M.Y., Al Suwaidi S.K.B.M., AlKatheeri R. (2020). Global epidemiology of ischemic heart disease: Results from the global burden of disease study. Cureus.

[4] Liu C.M., Shun C.T., Cheng Y.K. (1998). Soluble adhesion molecules and cytokines in perennial allergic rhinitis. Ann. Allergy Asthma Immun..

[5] Teng L.L., Shao L., Zhao Y.T., Yu X., Zhang D.F., Zhang H. (2010). The beneficial effect of n-3 polyunsaturated fatty acids on doxorubicin-induced chronic heart failure in rats. J. Int. Med. Res..

[6] Ferrara N., Gerber H.P. (1999). Vascular endothelial growth factor molecular and biological aspects. Curr. Top. Microbiol. Immunol..

[7] Nigam P.K. (2007). Biochemical markers of myocardial injury. Indian J. Clin. Biochem..

[8] Vasanthi H.R., ShriShriMal N., Das D.K. (2012). Phytochemicals from plants to combat cardiovascular disease. Curr. Med. Chem..

[9] Jagdale A.D., Kamble S.P., Nalawade M.L., Arvindekar A.U. (2015). Citronellol: A potential antioxidant and aldose reductase inhibitor from *Cymbopogon citratus*. Int. J. Pharm. Sci..

[10] Brito R.G., Guimarães A.G., Quintans J.S., Santos M.R., De Sousa D.P., Badaue-Passos D. (2012). Citronellol, a monoterpene alcohol, reduces nociceptive and inflammatory activities in rodents. J. Nat. Med..

[11] de Sousa D.P., Gonçalves J.C.R., Quintans-Júnior L., Cruz J.S., Araújo D.A.M., de Almeida R.N. (2006). Study of anticonvulsant effect of citronellol, a monoterpene alcohol, in rodents. Neurosci. Lett..

[12] Srinivasan S., Muruganathan U. (2016). Antidiabetic efficacy of citronellol, a citrus monoterpene by ameliorating the hepatic key enzymes of carbohydrate metabolism in streptozotocin-induced diabetic rats. Chem. Biol. Interact..

[13] Zhuang S.R., Chen S.L., Tsai J.H., Huang C.C., Wu T.C., Liu W.S., Tseng H.C., Lee H.S., Huang M.C., Shane G.T. (2009). Effect of citronellol and the Chinese medical herb complex on cellular immunity of cancer patients receiving chemotherapy/radiotherapy. Phytother. Res..

[14] Bastos J.F., Moreira Í.J., Ribeiro T.P., Medeiros I.A., Antoniolli A.R., De Sousa D.P. (2020). Hypotensive and vasorelaxant effects of citronellol, a monoterpene alcohol, in rats. Basic Clin. Pharmacol. Toxicol..

[15] Su Y.W., Chao S.H., Lee M.H., Ou T.Y., Tsai Y.C. (2010). Inhibitory effects of citronellol and geraniol on nitric oxide and prostaglandin E2 production in macrophages. Planta Med..

[16] De Menezes I.A.C., Moreira Í.J.A., De Paula J.W.A., Blank A.F., Antoniolli A.R., Quintans-Júnior L.J. (2010). Cardiovascular effects induced by *Cymbopogon winterianus* essential oil in rats: Involvement of calcium channels and vagal pathway. J. Pharm. Pharmacol..

[17] Tavares L.A., Rezende A.A., Santos J.L., Estevam C.S., Silva A.M., Schneider J.K. (2021). *Cymbopogon winterianus* essential oil attenuates bleomycin-induced pulmonary fibrosis in a murine model. Pharmaceutics.

[18] Swamy A.V., Wangikar U., Koti B., Thippeswamy A., Ronad P., Manjula D. (2011). Cardioprotective effect of ascorbic acid on doxorubicin-induced myocardial toxicity in rats. Indian J. Pharmacol..

[19] Marklund S., Marklund G. (1974). Involvement of the superoxide anion radical in the autoxidation of pyrogallol and a convenient assay for superoxide dismutase. Eur. J. Biochem..

[20] Aebi H., Catalase I. (1974). Methods in Enzymatic Analysis. Methods Enzym. Anal..

[21] Rotruck J.T., Pope A.L., Ganther H.E., Swanson A., Hafeman D.G., Hoekstra W.G. (1973). Biochemical role as a component of glutathione peroxidase. Science.

[22] Ellman G.L. (1959). Tissue sulfhydryl groups. Arch. Biochem. Biophys..

[23] Everaert B.R., Boulet G.A., Timmermans J.P., Vrints C.J. (2011). Importance of suitable reference gene selection for quantitative real-time PCR: Special reference to mouse myocardial infarction studies. PLoS ONE.

[24] Participants R.T. (1993). Coronary angioplasty versus coronary artery bypass surgery: The Randomized Intervention Treatment of Angina (RITA) trial. Lancet.

[25] Pezzuto J.M. (1997). Plant-derived anticancer agents. Biochem. Pharmacol..

[26] Boateng S., Sanborn T. (2013). Acute myocardial infarction. Dis. Mon..

[27] Elsherbiny N.M., Salama M.F., Said E., El-Sherbiny M., Al-Gayyar M.M. (2016). Crocin protects against doxorubicin-induced myocardial toxicity in rats through down-regulation of inflammatory and apoptic pathways. Chem. Biol. Interact..

[28] Ammar E.S.M., Said S.A., El-Damawary L., Suddek G.M. (2013). Cardioprotective effect of grape-seed proanthocyanidins on doxorubicin-induced cardiac toxicity in rats. Pharm. Biol..

[29] Swamy A.V., Gulliaya S., Thippeswamy A., Koti B.C., Manjula D.V. (2012). Cardioprotective effect of curcumin against doxorubicin-induced myocardial toxicity in albino rats. Indian J. Pharmacol..

[30] Barone F.C., Feuerstein G.Z. (1999). Inflammatory mediators and stroke: New opportunities for novel therapeutics. J. Cereb. Blood Flow Metab..

[31] Wang L., Wang L., Zhou X., Ruan G., Yang G. (2019). Qishen Yiqi dropping pills ameliorates doxorubicin-induced cardiotoxicity in mice via enhancement of cardiac angiogenesis. Med. Sci. Monit..

[32] Jones S.P., Greer J.J., Kakkar A.K., Ware P.D., Turnage R.H., Hicks M. (2004). Endothelial nitric oxide synthase overexpression attenuates myocardial reperfusion injury. Am. J. Physiol. Heart Circ. Physiol..

[33] Chen L.L., Yin H., Huang J. (2007). Inhibition of TGF-β1 signaling by eNOS gene transfer improves ventricular remodeling after myocardial infarction through angiogenesis and reduction of apoptosis. Cardiovasc. Pathol..

[34] Chen L.L., Zhu T.B., Yin H., Huang J., Wang L.S., Cao K.J. (2010). Inhibition of MAPK signaling by eNOS gene transfer improves ventricular remodeling after myocardial infarction through reduction of inflammation. Mol. Biol. Rep..

[35] Upadhyay S., Mantha A.K., Dhiman M. (2020). *Glycyrrhiza glabra* (Licorice) root extract attenuates doxorubicin-induced cardiotoxicity via alleviating oxidative stress and stabilising the cardiac health in H9c2 cardiomyocytes. J. Ethnopharmacol..

[36] Takano H., Hasegawa H., Zou Y., Komuro I. (2004). Pleiotropic actions of PPARg activators thiazolidinediones in cardiovascular diseases. Curr. Pharm. Des..

